# News and Coming Events

**Published:** 1907-08-31

**Authors:** 


### NEWS AND COMING EVENTS.

The Bradford Fever Hospital is to be improved by alterations and extensions at a cost of over £6,800. A new isolation pavilion and separate residential quarters for the nursing staff are provided for in the plans. The present administration block is to be enlarged by the addition of four rooms. The mortuary and outbuildings will also be enlarged.

At the annual meeting of subscribers of the Devonshire Hospital and Buxton Bath Charity, held at the Hospital on Tuesday, July 30 last, under the presidency of the Duke of Portland, it was stated that the year just passed has been a record one for the hospital, not only in the number of patients received (3,575) but also in the amount of subscriptions received. Since 1902 the hospital has been improved by the installation of a new *x*-ray apparatus, new fire and heating plant, and a completely new set of appliances, such as an arthromotor, radiant heat baths, and mechanical vibrators.

A gift of £500 has been made to the Bradford Royal Eye and Ear Hospital by Mrs. Alfred Illingworth, in memory of her father, Sir Isaac Holden. The present is the jubilee year of the Eye and Ear Hospital, in connection with which event the prefix of Royal was conferred on the institution recently by the King. Mrs. Illingworth's gift is to be used for the benefit of the Hospital and to perpetuate the memory of Sir Isaac Holden. The actual method of applying the donation has not yet been defined, but it will be decided when the Governors of the Hospital have had the opportunity of submitting their proposals to the donor.

Under the will of the late Dr. Nathaniel Rogers, the Senate of the University of London offer a prize of £100, open for competition to all members of the medical profession in the United Kingdom, for the best essay or dissertation setting forth the results of original investigations made by the candidate on any medical pathological subject during the preceding two years. Candidates will be permitted to present papers published during the preceding year as the dissertation. The essay or dissertation, by preference typewritten or printed, must be sent in not later than May 1, 1908, addressed to the Clerk of Committees, University of London, South Kensington.

In the current number of *The Crown* Dr. Hadwen gives an interesting account of the cancer curers in Wales, illustrated with a portrait of the brothers Evans. Dr. Hadwen relates pathetic stories of the credulity of their patients. " In one instance a case of sarcoma which several medical men had examined and declared to be inoperable, had, evidently owing to the irritating character of the application, taken on most rapid growth . . . but the sufferer, a very intelligent man, viewed this dangerous extension as an instance of the 'herb doctor's' skill. 'It is wonderful,' he said, 'how rapidly they are bringing it out!'" The "Secret Oil" which is used by the brothers is in all probability a mixture of which the active constituent is a strong solution of chloride of zinc. Its application is excruciatingly painful, and so far the results have been most discouraging. Dr. Hadwen concludes by remarking that the failure of the treatment has afforded the public "an object lesson which should teach some of them, perhaps, to keep their heads cool when the next butterfly of empiricism emerges from the local chrysalis into the light of publicity."

At the half-yearly meeting of the governors of the Pontypool and District Hospital, the executive reported that it had been decided to fit a complete electric installation at the hospital at a cost of £150.

Deaths from plague in India during the week ending July 13 last numbered 4,899, as compared with 5,492 during the preceding week. Of these 713 were in the Bombay Presidency, 3,692 in the Punjab, 146 in Burma, and 137 in Mysore. Deaths from the disease in Peshawar are reported to be steadily but slowly decreasing.

Merck's Annual Report.—The twentieth volume of this useful and interesting publication has just been issued, and is sent free to medical men and others interested in pharmacology on application to Mr. E. Merck, 16 Jewry Street, E.C. The book is the outcome of an honest desire to furnish a brief and impartial review of the therapeutic acquisitions of the last twelve months. It gives details, and in many cases full particulars, of the literature, of all the new remedies and drugs which have been placed on the market during the year 1906. The information regarding dosage, treatment, and chemistry of the various therapeutical novelties discussed is exceedingly interesting, and should prove useful to those who wish to try the new drugs. Especially worthy of notice are the articles dealing with recent serum therapy, and with such drugs as santonin and trypan red. The last-named compound has been found useful in cases of trypanosomiasis, and rejoices (if such an infliction can be enjoyed) in the awful name of "sodium-ortho-benzidine-mono-sulphoacid-dizazo-b-2-naphthylamine-3.6-sulpho-acid"!

The Street Arab.—Communal obligations, as Victor Hugo pointed out, are fulfilled by a few and neglected by the many, and in no case is this more true than with regard to the vagrant youth of the streets. The State takes care that the lad is educated to a certain degree and up to a certain age: after that it concerns itself solely in punishing, often with undue severity, the mistakes which its neglect of the school-leaving lad has made possible. After 14, the school-leaving age, the street gamin is thrown on his own resources, and it depends to a great extent on himself whether he becomes a respectable member of the community or a recruit to the army of our modern slum "Macaronis." Every poor lad at that stage is a potential hooligan, and with the exception of a few charitably disposed societies no one cares whether he becomes one in actuality. For the really destitute the homes founded by that great philanthropist and, let us add, true Imperialist, Dr. Barnardo, have done and are doing a work of salvation which is more clearly appreciated every day. For the self-dependent boy the Society of the Good Shepherd is doing as good work, and its annual report, just issued, shows how deserving of support that work is. The society has a home and a hostel, the former at Clapham, the latter at Camberwell Green. In the latter are lodged 50 homeless boys who are in daily employment in the city, while the home provides for 40 orphans, somewhat after the system in vogue at Stepney. Financial support to enable the society to rebuild the home is urgently needed, and an earnest appeal has been made by the Secretary on the generosity of the public. The Director and Treasurer of the home is Mr. Russell Baker, 4 Rectory Grove, Clapham, S.W., from whom all particulars may be obtained, and to whom subscriptions may be forwarded in aid of the excellent work which the society has so unostentatiously been doing during the last six years.

